# ﻿First report of the genus *Tenuibaetis* (Ephemeroptera, Baetidae) from Thailand revealing a complex of cryptic species

**DOI:** 10.3897/zookeys.1084.78405

**Published:** 2022-02-01

**Authors:** Chanaporn Suttinun, Jean-Luc Gattolliat, Boonsatien Boonsoong

**Affiliations:** 1 Animal Systematics and Ecology Speciality Research Unit (ASESRU), Department of Zoology, Faculty of Science, Kasetsart University, Bangkok 10900, Thailand Kasetsart University Bangkok Thailand; 2 Museum of Zoology, Palais de Rumine, Place Riponne 6, CH-1005 Lausanne, Switzerland Museum of Zoology Lausanne Switzerland; 3 University of Lausanne (UNIL), Department of Ecology and Evolution, CH-1015 Lausanne, Switzerland University of Lausanne Lausanne Switzerland

**Keywords:** COI, mayflies, MOTUs, new species, Southeast Asia, *Tenuibaetispanhai* sp. nov.

## Abstract

A new species of the genus *Tenuibaetis* Kang & Yang, 1994 is described from Thailand and the genus is reported for the first time from this country. *Tenuibaetispanhai***sp. nov.** is easily distinguished from other known *Tenuibaetis* by the complete absence of hindwing pads. Molecular evidence based on COI confirmed the validity of the new species. Additional putative species of *Tenuibaetis* based on molecular evidence only are considered as Molecular Operational Taxonomic Units (MOTUs) without description. The morphological characters of the new species and its closely related species are discussed; a key to the Oriental species is provided.

## ﻿Introduction

Kang et al. (1994) established the subgenus Tenuibaetis, and Baetis (Tenuibaetis) pseudofrequentus Müller-Liebenau, 1985 from Taiwan was considered as the type species. This subgenus originally included three species (B. (T.) pseudofrequentus Müller-Liebenau, 1985, B. (T.) arduus Kang & Yang, 1994 and B. (T.) inornatus Kang & Yang, 1994) and was characterized by the following larval characters: mandibles with margin between prostheca and mola without setae, spines or serration, apex of labial palps somewhat acute, femoral villopore present and paraproct with a patch of notched scales. Waltz and McCafferty (1997) synonymized *Tenuibaetis* with *Baetiella* based on the shape of the labial palp. Fujitani et al. (2003b, 2011) questioned this transfer by stating that the larvae of *Tenuibaetis*, significantly differ from those of *Baetiella* and related genera by the inner margins of cerci fringed with setae in *Tenuibaetis* but glabrous in *Baetiella*, and the robust setae with medial ridges on the dorsomedian surface of the larval femur. Therefore, they revalidated *Tenuibaetis* and raised it to the generic rank.

The genus *Tenuibaetis* currently contains seven species: *T.flexifemora* (Gose, 1980) from Japan (Gose 1980); *T.pseudofrequentus* (Müller-Liebenau, 1985) from Taiwan, Japan and Hong Kong (Müller-Liebenau 1985; Tong and Dudgeon 2000; Fujitani et al. 2003a, 2003b, 2011); *T.frequentus* (Müller-Liebenau & Hubbard, 1985) from Sri Lanka and India (Müller-Liebenau and Hubbard 1985; Balaji et al. 1990; Sivaramakrishman and Venkataraman 1990; Kubendran et al. 2015); *T.arduus* (Kang & Yang, 1994) and *T.inornatus* (Kang & Yang, 1994) from Taiwan (Kang et al. 1994), *T.parviptera* Fujitani, 2011 from Japan (Fujitani et al. 2011), and *T.fujitanii* Kaltenbach & Gattolliat, 2019 from Indonesia (Kaltenbach and Gattolliat 2019). Two additional species, *Baetisursinus* Kazlauskas, 1963 and *B.hissaricus* Novikova, 1991, were considered to belong to this genus by Kluge (2021), but they were never formally transferred to this genus. The distribution of this genus is encompassing the whole oriental realm and the most Eastern part of Palearctic realm.

In the last decade, knowledge of the diversity of the Baetidae in Thailand has grown, as seven genera were reported for the first time from this area: *Procloeon* Bengtsson, 1915 (Tungpairojwong and Bae 2015; Kluge 2016), *Anafroptilum* Kluge, 2012 (Kluge and Novikova 2017), *Platybaetis* Müller-Liebenau, 1980 (Sutthinun et al. 2018), *Centroptella* Braasch & Soldán, 1980 (Kluge et al. 2020), *Indocloeon* Müller-Liebenau, 1982 (Kluge and Suttinun 2020), and *Procerobaetis* Kaltenbach & Gattolliat, 2019 (Suttinun et al. 2021). The newest genus, *Cymbalcloeon* Suttinun, Gattolliat & Boonsoong, 2020, is for the moment only known from Thailand (Suttinun et al. 2020). We describe a new species of *Tenuibaetis* from Thailand, based on material collected during the first mass survey of the family Baetidae in this country (Suttinun 2021). Additionally, we also present cryptic diversity within this genus treated as Molecular Operational Taxonomic Units (MOTUs) based on molecular evidence only (COI), without formal description of the species (Floyd et al. 2002; Blaxter et al. 2005; Morard et al. 2016; Kaltenbach et al. 2020). As Thailand is in the middle of the Oriental realm, our study will provide a better understanding of the distribution of this genus.

## ﻿Materials and methods

The specimens were collected from headwater streams in different parts of North, West and South of Thailand (Table 1, GPS map versatile navigator (Garmin eTrex 10)). They are preserved in 95% ethanol. Larval dissection was performed in Cellosolve, with subsequent mounting on slides with Euparal. Measurements (given in mm) and photographs were taken using a Visionary LK System (Dun, Inc., USA). All drawings were made with a camera lucida attached to a compound microscope and scanned for editing in Procreate 5X (iOS application). Final plates were prepared with Adobe Photoshop CC 2020.

**Table 1.** GPS coordinates of locations of examined specimens.

Species	Locality	GPS coordinates
*T.panhai* sp. nov.	Kanchanaburi (KN)	14°34'57.9"N, 98°34'52.0"E
		14°33'10.8"N, 98°33'94.3"E
		14°58'21.0"N, 98°53'50.3"E
	Ratchaburi (RB)	13°31'45.6"N, 99°14'65.6"E
	Petchaburi (PC)	12°38'14.5"N, 99°30'59.5"E
	Loei (LE)	17°06'40.7"N, 101°28'72.0"E
	Chiang Rai (CR)	19°51'76.8"N, 99°39'07.8"E
		20°03'15.8"N, 99°49'28.2"E
		20°05'36.0"N, 99°46'79.7"E

DNA was extracted using non-destructive methods to allow subsequent morphological analysis (see Vuataz et al. 2011 for details). Part of the COI (a 658 bp fragment of the mitochondrial gene cytochrome oxidase subunit 1) was amplified using the primers LCO1490 and HCO2198 (Folmer et al. 1994). The polymerase chain reaction (PCR) conditions and procedure were performed as described by Kaltenbach et al. (2020). Sequencing was done using Sanger’s method (Sanger et al. 1977). The genetic distances between species were calculated using Kimura 2-parameter distances (K2P, Kimura 1980), using MEGA X (Kumar et al. 2018). Sequence alignment and editing were performed using ClustalW. The phylogenetic tree was analysed by Bayesian inference using MrBayes. The best evolution model obtained was Hasegawa-Kishino-Yano and proportion of invariable sites (HKY+I). The GenBank accession numbers are given in Table 2, nomenclature of gene sequences follows Chakrabarty et al. (2013). Other analyzed *Tenuibaetis* sequences were obtained from GenBank: *T.frequentus* (LC056074.1) and *T.flexifemora* (KX824012.1; KP970712.1). *Liebebiellavera* (LC056071.1) was used as an outgroup. The nomenclature used for Molecular Operational Taxonomic Units (MOTUs) broadly follows Morard et al. (2016) original proposal.

**Table 2.** Sequenced specimens of *Tenuibaetis*.

Species	Locality	Code	Genbank #	GenSeq Nomenclature
*T.panhai* sp. nov.	Kanchanaburi	TEKN01	OM264189	genseq-1 COI
		TEKN06	OM319584	genseq-3 COI
	Ratchaburi	TERB01	OM302269	genseq-3 COI
	Petchaburi	TEPC02	OM302305	genseq-3 COI
		TEPC03	OM319569	genseq-3 COI
	Loei	TELE01	OM302308	genseq-3 COI
		TELE02	OM303507	genseq-3 COI
	Chiang Rai	TECR01	OM302358	genseq-3 COI
		TECR02	OM303508	genseq-3 COI
T.cf.panhai sp. I	Patthaluang	TEPT01	OM320557	genseq-4 COI
	Nakhon Sri Thammarat	TENT01	OM320559	genseq-4 COI
	Surat Thani	TEST01	OM320558	genseq-4 COI
	Narathiwat	TENW01	OM320563	genseq-4 COI
T.cf.panhai sp. II	Chiang Mai	TECM02	OM320576	genseq-4 COI
		TECM03	OM320587	genseq-4 COI
		TECM04	OM320571	genseq-4 COI
		TECM05	OM320562	genseq-4 COI
*T.frequentus*	India		LC056074	–
*T.flexifemorus*	Japan		KX824012	–
			KP970712	–

The distribution map was generated with the SimpleMappr software (https://simplemappr.net; Shorthouse 2010).

The material was deposited in the collection of the Zoological Museum at Kasetsart University in Bangkok, Thailand (**ZMKU**) and at the Museum of Zoology in Lausanne, Switzerland (**MZL**).

We followed all guidelines of the Animal Ethics Committee of Kasetsart University (approval no. ACKU61-SCI-029) for collecting the mayfly specimens.

## ﻿Taxonomy

### ﻿ Tenuibaetis panhai sp. nov.

Taxon classificationAnimaliaEphemeropteraBaetidae

F7220416-C4E6-5B9A-9306-7F2462784DCF

http://zoobank.org/B39C17B1-A135-4DEC-8172-CB6C497F89AD

Figs 1 , 2 , 3 , 4

#### Type material.

***Holotype*.** Thailand • larva; Kanchanaburi, Thong Pha Phumi District, Pra Chum Mai; 14°34'58"N, 98°34'52"E; 269 m; 31 Jan. 2019; leg. C. Suttinun; on slide; Genbank OM264189; TEKN01; ZMKU. ***Paratypes*.** Thailand • 7 larvae; same data as holotype; 1 on slide TEKN03; 4 in alcohol; ZMKU; 1 on slide GBIFCH00829251; 1 in alcohol; TEKN02; GBIFCH00673241; MZL. ***Other material*.** Thailand • 1 larva; Kanchanaburi, Thong Pha Phumi District, Pat Sadu Klang; 14°33'11"N, 98°33'94"E; 349 m; 1 Feb. 2019; leg. C. Suttinun; in alcohol; ZMKU. • 6 larvae; Kanchanaburi, Thong Pha Phumi District, Huai Pak Kok; 14°39'57"N, 98°32'04"E; 175 m; 1 Feb. 2019; leg. C. Suttinun; in alcohol; ZMKU. • 2 larvae; Kanchanaburi, Thong Pha Phumi District, Huai Pheung Ban Lung Yee; 14°58'21"N, 98°53'50"E; 709 m; 1 Feb. 2018; leg. C. Auychinda; 1 in alcohol (mouthpart); Genbank OM319584; TEKN05; TEKN06; ZMKU. • 1 larva; Ratchaburi, Suan Phueng District, Bo Klueng; 13°31'46"N, 99°14'66"E; 180 m; 25 Nov. 2018; leg. C. Suttinun; in alcohol (mouthpart); Genbank OM302269; TERB01; ZMKU. • 5 larvae; Petchaburi, Kaeng Krachan District, Huai Sat Lek; 12°38'15"N, 99°30'60"E; 166 m; 25 Feb. 2018; leg. C. Suttinun; 4 in alcohol; 1 on slide; Genbank OM302305, OM319569; TEPC02; ZMKU. • 14 larvae; Loei, Phu Luang District, Ban Non Pattana; 17°06'41"N, 101°28'72"E; 527 m; 18 Dec. 2018; leg. C. Suttinun; 10 in alcohol; 3 on slides; Genbank OM302308, OM303507; TELE01; TELE03; TELE04; ZMKU; 1 on slide; TELE02; GBIFCH00829259; MZL • 2 larvae; Chiang Rai, Mueng District, Mae Korn Stream; 19°51'77"N, 99°39'08"E; 534 m; 6 May. 2019; leg. C. Suttinun; in alcohol; ZMKU. • 2 larvae; Chiang Rai, Mueng District, Nang Lae Nai waterfall; 20°03'16"N, 99°49'28"E; 529 m; 7 May. 2019; leg. C. Suttinun; 1 in alcohol; 1 on slide; Genbank OM303508; TECR02; ZMKU. • 3 larvae; Chiang Rai, Mae Chan District, Huai Kang Pla waterfall; 20°05'36"N, 99°46'80"E; 519 m; 7 May. 2019; leg. C. Suttinun; 2 in alcohol; 1 on slide; Genbank OM302358; TECR01; ZMKU.

#### Description.

**Figure 1.**
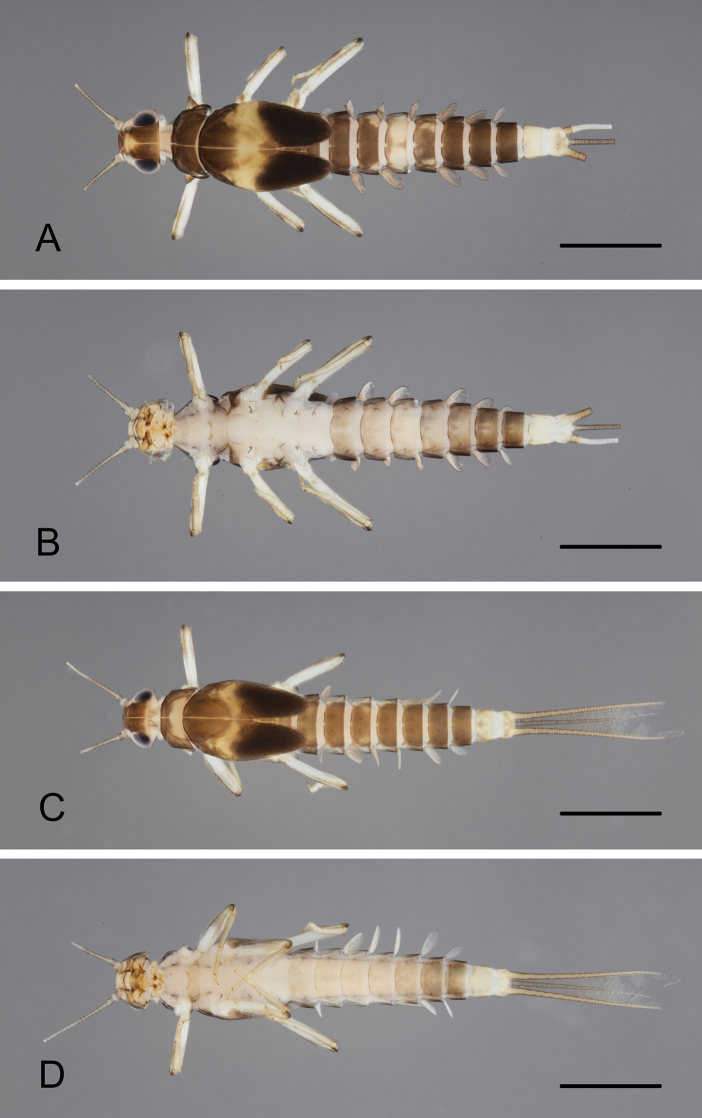
*Tenuibaetispanhai* sp. nov., larval habitus. Kanchanaburi province **A** dorsal view **B** ventral view; Loei province: **C** dorsal view **D** ventral view. Scale bar: 1 mm.

***Coloration*** (Fig. 1). Head dorsally brown and yellow, with a yellow marking between ocelli. Thorax dorsally brown, pronotum with (Fig. 1C) or without (Fig. 1A) posterior yellow marking; mesonotum medially with a yellow transverse band. Abdomen dorsally brown; tergite III with (Fig. 1A) or without (Fig. 1C) a pair of yellow markings on lateral sides; tergite IV yellowish with or without median brown marking; tergite V with or without anterior yellow marking; tergite VIII with or without posterior yellow marking; tergites IX–X yellow. Head and thorax ventrally whitish; abdomen ventrally light brown; sternites VI–VIII darker brown; sternites IX–X yellow. Legs light brown; dorsal, ventral, and apical femur margins darker brown with brown stripes distomedially; claws distally dark brown. Caudal filaments light brown without darker band or pattern.

**Head. *Antenna*.** Flagellum with lanceolate spines at apex of each segment.

**Figure 2.**
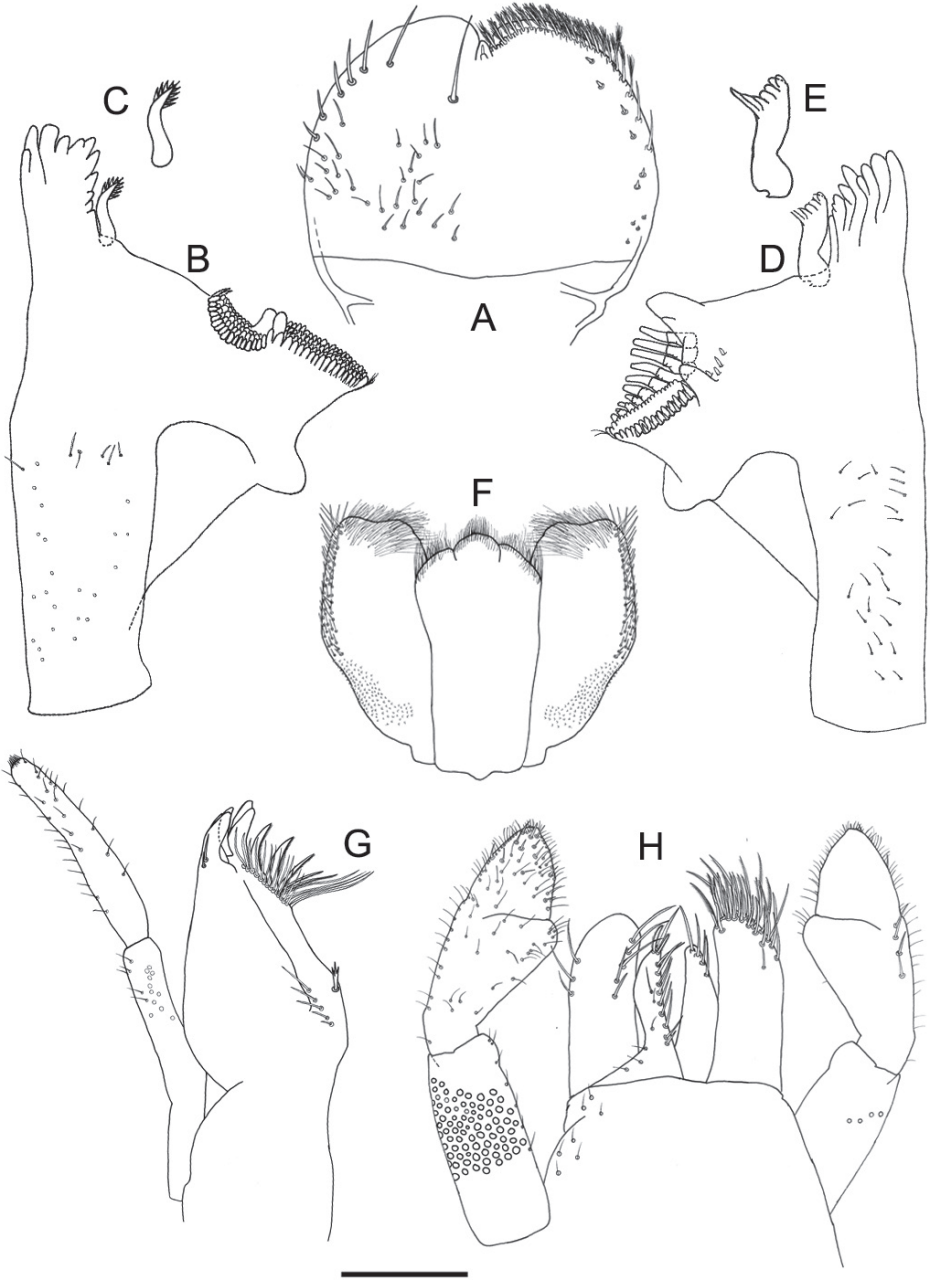
*Tenuibaetispanhai* sp. nov., larval morphology **A** labrum **B** right mandible **C** right prostheca **D** left mandible **E** left prostheca **F** hypopharynx **G** maxilla **H** labium. Scale bar: 0.1 mm.

***Labrum*** (Fig. 2A). Subrounded, length 0.66–0.74 × maximum width. Distal margin with medial emargination. Dorsally with submarginal arc composed of one long, pointed, simple seta medially plus two long, pointed, simple setae laterally and four long, pointed, simple setae decreasing in size along margin; dorsal surface with short, fine, simple setae scattered medially toward the basal area. Ventrally with submarginal row of setae composed of about 20 lateral long, feathery setae equal in size and a row of stout, simple setae laterally near margin.

***Right mandible*** (Fig. 2B, C). Incisors fused. Outer set with 4 denticles composed of two pointed denticles plus one larger, blunt denticle and one pointed denticle; inner sets with 4 pointed denticles; each denticle separated by a deep groove. Inner margin of innermost denticle with a row of minute teeth. Prostheca robust, apicolaterally denticulate. Margin between prostheca and mola straight, without setae. Tuft of setae at apex of mola present.

***Left mandible*** (Fig. 2D, E). Incisors fused. Outer and inner sets of pointed denticles with 3 + 3 denticles; each denticle separated by a deep groove, plus a minute intermediate denticle between sets. Inner margin of innermost denticle with minute denticles. Prostheca slightly shorter than incisor, robust, apically denticulate, with a comb-shaped structure. Margin between prostheca and mola straight without setae. Tuft of spine-like setae absent at base of mola. Subtriangular process long and wide, above level of area between prostheca and mola. Denticles of mola apically as wide as basal. Setae present at apex of mola.

Both mandibles with lateral margin almost straight. Basal half with fine, simple setae scattered over dorsal surface.

***Hypopharynx*** (Fig. 2F). Lingua slightly shorter than superlingua, longer than broad, with medial tuft of long, thin setae. Superlingua distally with a concave margin, with long, fine setae along distal margin; lateral margin rounded with simple setae along lateral margin.

***Maxilla*** (Fig. 2G). Galea-lacinia with two long, fine, simple setae under crown. Inner dorsal row of setae with three denti-setae; distal denti-seta tooth-like, middle denti-seta slender and pectinate, proximal denti-seta very long, slender, simple setae. Medially with one trifid, stout seta and five short to long, simple setae. Maxillary palp 1.4–1.5 × as long as length of galea-lacinia, 2-segmented; fine, simple setae scattered over surface of maxillary palp. Palp segment II 1.3 × length of segment I. Apex of last segment conical.

***Labium*** (Fig. 2H). Glossae basally broad, narrowing toward apex, shorter than paraglossae; inner margin with nine long, simple setae; apex with one long, simple seta and two medium, robust, pectinate setae; outer margin with four long, simple setae; dorsal surface with a long, simple seta medially; basal area with fine scattered setae. Paraglossae sub-rectangular, apically rounded, with three rows of setae, distal row of very long, pectinate, simple setae, other rows of pectinate long and medium setae; one curved, blunt, simple seta at inner apical margin; two long, simple setae in outer margin near three rows of setae; dorsal surface with one medium, simple seta anteromedially; dorsally with row of five long, simple setae parallel to inner margin, with an arc of three long, simple setae at outer margin; basal area with medium, spine-like setae scattered. Labial palp with segment I 0.8 × length of segments II and III combined. Segment I covered with micropores and few fine, simple setae. Segment II with poorly developed, apically rounded, distomedial protuberance; tuft of medium, fine, simple setae present at apex of protuberance; inner margin with medium, fine, simple setae; outer margin with short, fine, simple setae; dorsally with medium, fine, simple, scattered setae; dorsally with row of 4–6 medium, simple setae. Segment III conical, slightly asymmetrical; length subequal to width; covered with medium simple setae and stout simple setae anterolaterally.

**Figure 3.**
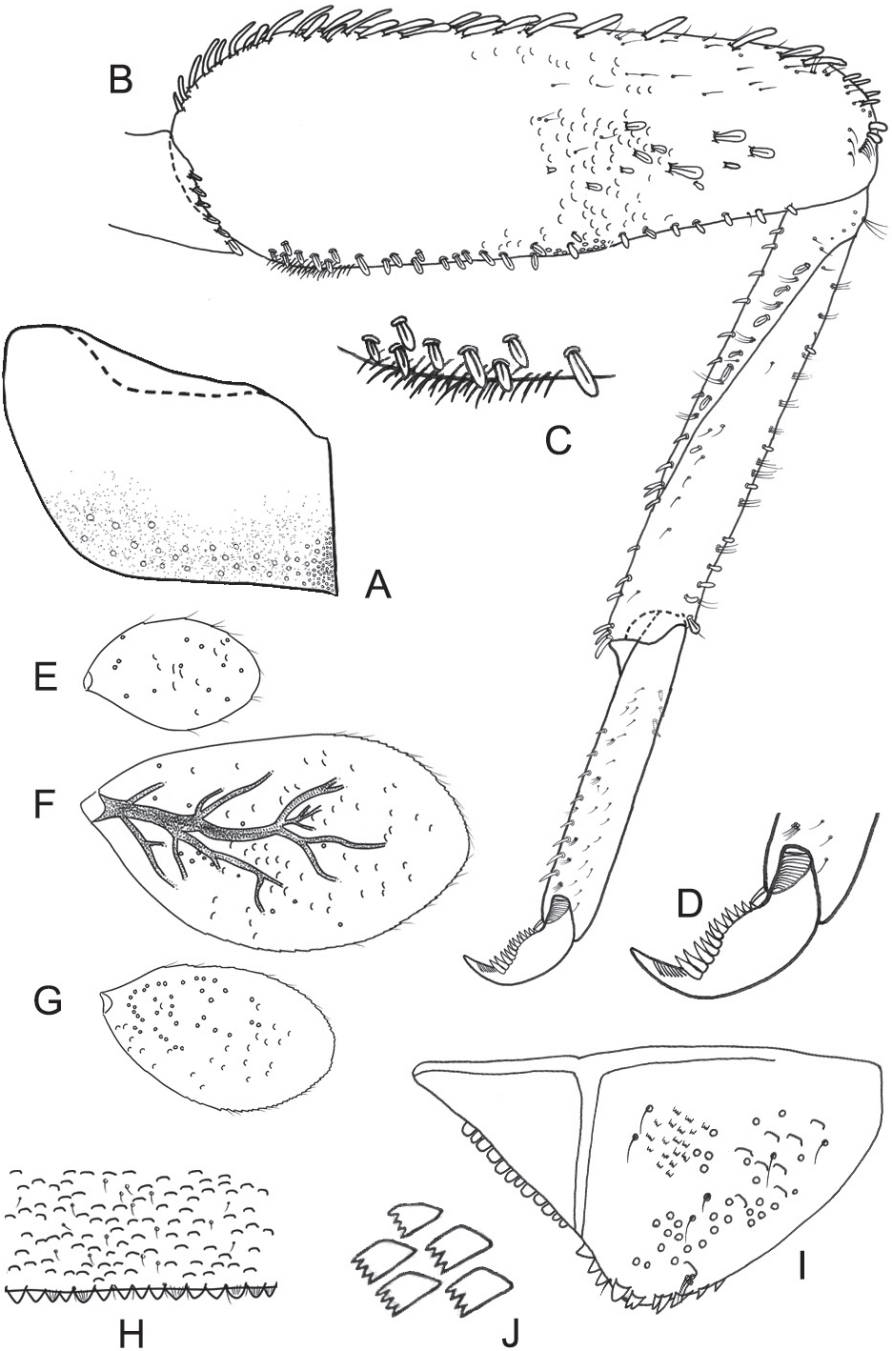
*Tenuibaetispanhai* sp. nov., larval morphology **A** metathorax without hindwing pad **B** foreleg **C** femoral patch **D** claw **E** gill I **F** gill V **G** gill VII **H** distal margin of tergite IV **I** paraproct **J** notched scales on paraproct.

**Thorax. *Hindwing pads*** (Fig. 3A). Absent.

***Foreleg*** (Fig. 3B–D). Ratio of foreleg segments 2.1:1.5:1:0.4. ***Femur*.** Length 2.9 × maximum width; dorsal margin with a row of 18–25 apically rounded, simple setae; length of setae 0.2 × maximum width of femur; anterior surface with 5–10 spatulate setae medially and about 28 acute, lanceolate setae close to ventral margin; apex rounded, with one pair of apically rounded, simple seta and two rows of stout, apically rounded, simple setae along apical margin; posterior surface with one row of stout, spatulate setae transverse anteromedially; femoral patch strongly developed. ***Tibia*.** Dorsal margin with a few short, spine-like setae and a pair of short, spine-like seta apically; ventral margin with a row of 7–13 acute, spine-like, curved setae and three long, spine-like apical setae; tibio-patella suture on basal 2/3 area with a row of eight stout, spatulate setae along suture. ***Tarsus*** (Fig. 3B, C). Dorsal margin nearly bare, with a few acute, simple setae on proximal area; ventral margin with one row of acute, curved, spine-like setae increasing apically; apex with one short, spine-like seta; claw curved, apically pointed, with one row of 11–13 denticles increasing apically; subapical setae absent.

**Abdomen. *Terga*** (Fig. 3H). Surface with scattered scales or scale-bases and micropores. Posterior margin of terga with row of apically, blunt, triangular spines.

***Gills*** (Fig. 3E–G). Present on segments I–VII; oval shaped. Margins serrate with small spines. Tracheation (Fig. 3F) extending from main trunk to inner and outer margins. Gill I (Fig. 3E) reduced, 0.3 × length of segment II; gills II–VI 1.2 × length of following segment; gill VII (Fig. 3G) 0.8 × length of segment VIII.

***Paraproct*** (Fig. 3I, J). Posterior margin with 5–7 pointed spines; surface with U-shaped scale base, micropores and fine, simple setae, and with a patch of notched scales (Fig. 3J); posterolateral extension (cercotractor) with 9–12 marginal spines.

***Caudal filaments*** (Fig. 1). Cerci ca. 0.5 × body length. Paracercus ca. 0.4 × body length.

#### Diagnostic characters.

**Larva.** The main diagnosis character is the absence of hindwing pads, followed by a combination of characters: A) distinct pattern on thorax and abdomen or “Zebra form,” as in Fig. 1; B) labrum dorsal submarginal arc composed of one long, pointed, simple seta medially plus two long, pointed, simple setae laterally and four long, pointed, simple setae decreasing in size along margin; C) right mandible: incisors with 4 + 4 pointed denticles, each denticle separated by a deep groove; D) left mandible: incisors with 3 + 3 pointed denticles plus a minute intermediate denticle between sets; E) hypopharynx: lingua with medial tuft of long, fine setae; superlingua lateral margin with long, simple setae; F) maxillary palp longer than galea-lacinia, apex conical; G) femur: dorsal margin with 15–25 apical rounded, simple seta, anterior surface with 5–10 spatulate setae; H) claw with a row of 11–13 denticles; I) paraproct: distal margin with 5–7 spines, surface with a patch of notched scales.

#### Winged stages.

Unknown.

#### Etymology.

*Tenuibaetispanhai* sp. nov. is dedicated to Professor Dr. Somsak Panha (Animal Systematics Research Unit, Department of Biology, Faculty of Science, Chulalongkorn University, Bangkok, Thailand) for his outstanding contributions to the systematics study of the fauna in Thailand.

#### Distribution.

Kanchanaburi (KN), Ratchaburi (RB), Petchaburi (PC), Chiang Rai (CR), and Loei (LE) provinces of Thailand.

#### Biological aspects.

**Figure 4.**
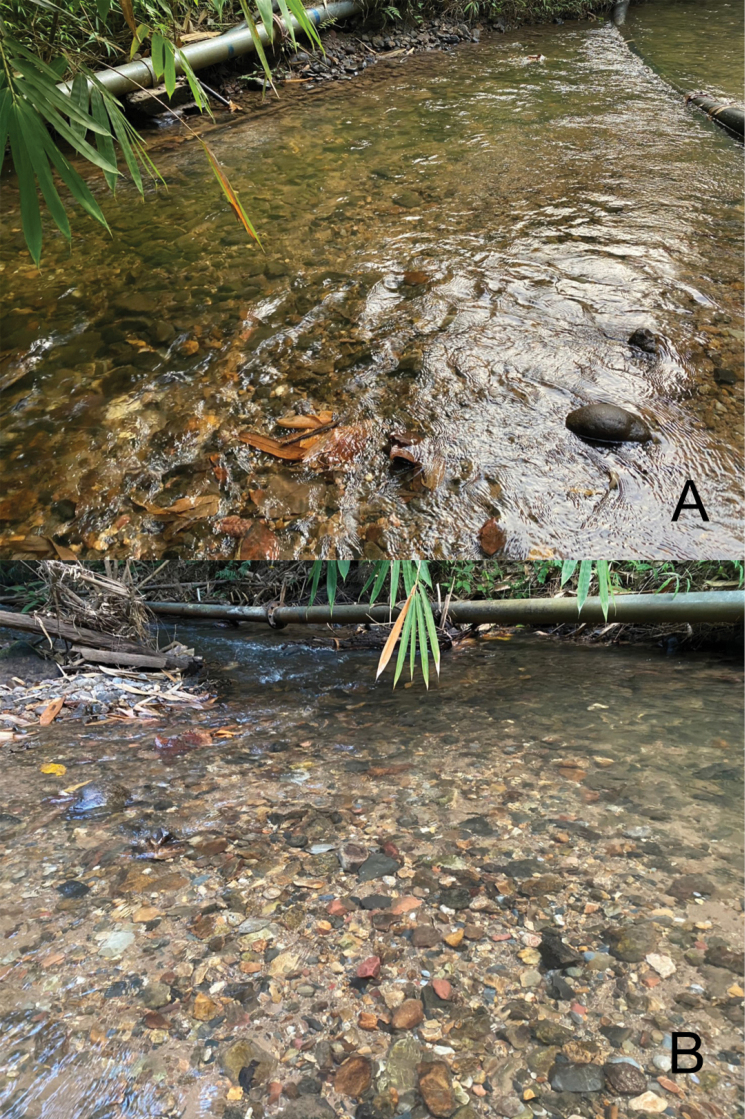
Type locality and larval habitats of *Tenuibaetispanhai* sp. nov. **A, B** fast-flowing water with bottom sand, pebble and gravel (Pa Chum Mai, Mae Klong headwater stream).

The specimens were collected in headwater streams (Fig. 4A) and above waterfalls at different altitudes (150–700 m a.s.l.). The streams were mostly located in forest areas with a partly complete canopy; the substrate was dominated by pebbles, gravel, and sand. The larvae were found on the undersides of pebbles in fast-flowing water (Fig. 4B). The waterfalls were located in areas with human disturbing activity as tourist attractions. They were collected together with other mayfly species: *Cymbalcloeonsartorii* Suttinun, Gattolliat & Boonsatien, 2020 (Baetidae), *Liebebiellavera* (Baetidae), and *Afronurus* spp. (Heptageniidae).

#### Molecular analysis.

COI sequences were obtained from specimens for each locality (Table 2). The K2P analysis revealed interspecific distances between *T.panhai* sp. nov. and the available *Tenuibaetis* species ranging between 17% and 27% (Table 3). The intraspecific distance was very limited within the nine sequences of *T.panhai* sp. nov. (0% to 4%).

**Table 3.** Genetic distances (COI) between sequenced specimens and MOTUs, using the Kimura 2-parameter.

Species		1	2	3	4	5
1	*T.panhai* sp. nov.	0.00–0.05				
2	T.cf.panhai sp. I	0.15–0.19	0.00–0.03			
3	T.cf.panhai sp. II	0.18–0.20	0.22–0.24	0.00		
4	*T.frequentus*	0.16–0.19	0.18–0.19	0.16	–	
5	*T.flexifemora*	0.24–0.27	0.24–0.26	0.23	0.23	0.00

Sequences of eight specimens, morphologically indistinct from *T.panhai* sp. nov. present genetic distance ranging between 15% and 20% to *T.panhai* sp. nov. These eight sequences are separated into two distinct groups. To depict the genetic diversity of *Tenuibaetis* in Thailand, we propose to consider these two groups as Molecular Operational Taxonomic Units (MOTUs) corresponding respectively to T.cf.panhai sp. I (Southern) and T.cf.panhai sp. II (Chiang Dao), based on genetic evidence only (COI; Table 2). The K2P distances of T.cf.panhai sp. I and T.cf.panhai sp. II range between 22% and 24% (Table 3). The intraspecific distances within MOTUs are limited (0% to 3%).

**Figure 5.**
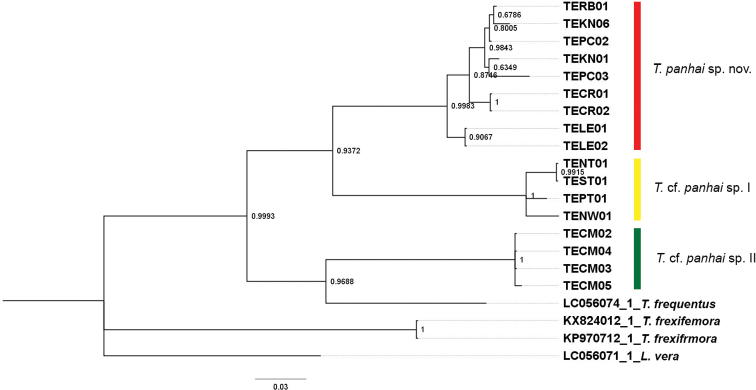
The Bayesian COI reconstruction of *Tenuibaetis* from the Oriental region. *Tenuibaetispanhai* sp. nov. (Red). T.cf.panhai sp. I (Yellow). T.cf.panhai sp. II (Green). *Liebebiellavera* (genbank accession no. LC056071) as an outgroup. The posterior probability was represented for each node.

The COI reconstruction was built by the Bayesian Interference (BI) using MrBayes (Fig. 5). Seventeen sequences of *Tenuibaetis* in Thailand are separated into two main distinct clades: the first clade includes *T.panhai* sp.nov. and T.cf.panhai sp. I while the second clade includes T.cf.panhai sp. II and *T.frequentus*.

## ﻿Discussion

*Tenuibaetispanhai* sp. nov. belongs to the genus *Tenuibaetis* based on the following characters defined by Kang et al. (1994) and Fujitani et al. (2003): mandible with a margin between the mola and prostheca without setae; a pointed apex of labial palp segment III, with segment II poorly expanded at the inner distal margin; villopore on the anteromedial corner of each femur; paraproct with a patch of notched scales medially; and robust setae with median ridge on the dorsomedian surface of the larval femur. The new species can be easily distinguished from all the other species of *Tenuibaetis* by the lack of hindwing pads. The combinations of characters commonly used to differentiate Oriental species of *Tenuibaetis* (Kaltenbach and Gattolliat 2019) are listed in Table 4. A comparison between the new species and the other known species of *Tenuibaetis* indicates a close morphological similarity between the new species and *T.flexifemora* (from Japan) in terms of the dorsal pattern coloration, the ratio of the length vs. the width of labrum, the shape of the spines on the distal margin of terga, the ratio of the length of gill IV to gill I, and the ratio of the terminal filament to the cerci. The new species also shows similarities with *T.frequentus* (from Sri Lanka, and India) regarding the dorsal pattern coloration, the ratio of the length vs. width of the labrum and the lack of patterning, and the ratio of the length of the maxillary palp versus the galea-lacinia.

**Table 4.** Larval character states of Oriental *Tenuibaetis* species (modified from Table 1 in Kaltenbach and Gattolliat 2019, p. 21).

		*T.panhai* sp. nov.	*T.fujitanii*	*T.pseudofrequentus*	*T.arduus*	*T.inornatus*	*T.frequentus*
**Colouration**	Dorsal pattern	distinct pattern	rather uniform brown	distinct pattern	distinct pattern	distinct pattern	distinct pattern
		(Figs 1–2 in this study)	(fig. 1a in Kaltenbach and Gattolliat 2019)	(fig. 9 in Müller-Liebenau 1985)	(fig. 27 in Kang et al. 1994)	(figs 12, 26 in Kang et al. 1994)	(fig. 10 in Müller-Liebenau and Hubbard 1985; fig. 1 in Kubendran et al. 2015)
**Labrum**	Length vs. width	0.7×	0.7×	0.8×	0.8×	0.8×	0.7×
	Pattern	absent	absent	Absent	absent	U-shaped dark marking	absent
**Maxillary palp**	Length vs. galea-lacinia	1.45×	1.1×	1.3×	1.2×	1.15×	1.4×
**Forefemur**	Number of dorsal setae	15–23	19–24	about 14	about 13	?	about 15
**Terga**	Spines at posterior margin	triangular, blunt; wider than long or about as wide as long	mostly rounded; wider than long	triangular, pointed; longer than wide	triangular, blunt; wider than long	triangular, blunt; wider than long	triangular, pointed; longer than wide
**Gills**	Tracheation	distinct, till margins	basal part of trunk	Obscure	obscure	distinct, till margins	obscure
	Length Gill IV / Gill I	2.7×	2.3×	2.7×–3.1×	2.3×	1.5×	2.0×
**Paraproct**	Number of marginal spines	5–7	about 15	about 10	about 14	about 11	about 20
**Terminal filament**	Length paracerus vs. cerci	0.7×	0.7×–0.8×	0.5×–0.6×	0.76×	0.65×	0.6×
**Distribution**		Thailand	Indonesia	Taiwan	Taiwan	Taiwan	Taiwan, Sri lanka, India
**References**		Present study	Kaltenbach and Gattolliat (2019)	Müller-Liebenau (1985)	Kang et al. (1994)	Kang et al. (1994)	Müller-Liebenau and Hubbard (1985); Kubendran et al. (2015)

The genetic distances between the new species and MOTUs are unexpected, with a range between 15% and 20% (K2P, Table 3), which is similar to the interspecific distance between the available *Tenuibaetis* species. Ball et al. (2005) also reported in a few cases a mean interspecific distance of 18% for congeneric mayflies in the USA and Canada. The intraspecific distances of each new species (including MOTUs) are very low, as expected, ranging from 0% to 4% (K2P). MOTUs were used for mayflies of the genus *Labiobaetis* from the Phillipines in Kaltenbach et al. (2020). This approach was originally defined and used to solve the enormous diversity of small organisms like nematodes and foraminifera (Floyd et al. 2002; Blaxter et al. 2005; Morard et al. 2016). All identified MOTUs of *Tenuibaetis* of Thailand are morphologically indistinct from *T.panhai* sp. nov., but present all the differences with the other known species of *Tenuibaetis* especially the lack of hindwing pads. We may assume the geographical and ecological factors to be the main drivers of the molecular evolution as T.cf.panhai sp. I is distributed in South of Thailand only (allopatric distribution) while T.cf.panhai sp. II was only collected in a waterfall from Chiang Dao Mountain Range, Chiang Mai province. Additional material and investigations will be necessary to confirm their status in the future. Because of the interspecific genetic distance between MOTUs and *T.panhai* sp. nov., but without morphological support, T.cf.panhai sp. I and T.cf.panhai sp. II remain considered as species hypotheses for now without further treatment in this paper.

In conclusion, the genus *Tenuibaetis* is widespread and common in Thailand. Due to its pattern (the “Zebra form”), it can be easily recognized even in the field. The distribution should be used for taxa delimitation. However, definitive species attributions of additional populations will require molecular confirmation. We propose two MOTUs; they will be considered or not as valid species in the future.

**Figure 6.**
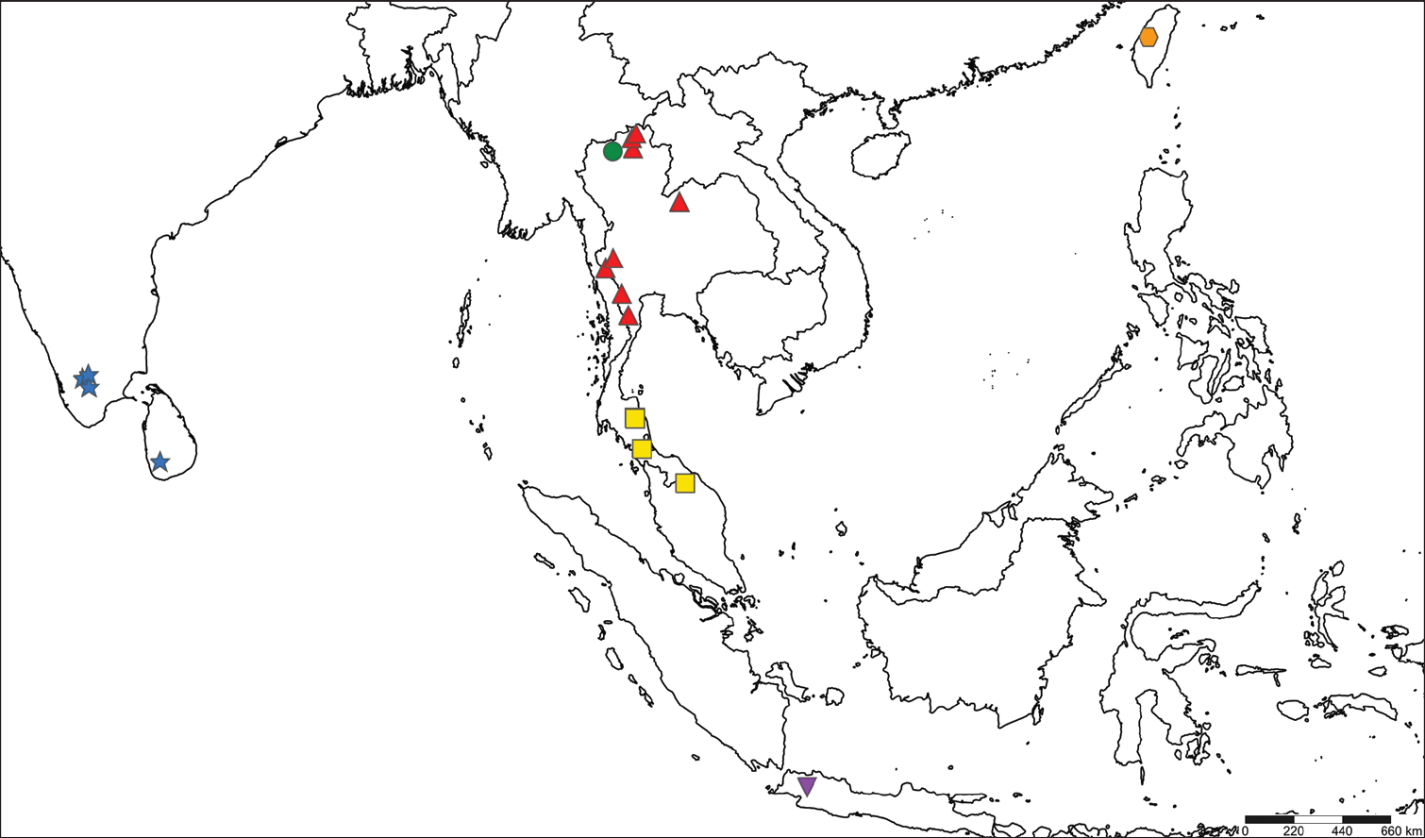
Distribution of the genus *Tenuibaetis* in the Oriental region. *Tenuibaetispanhai* sp. nov. (Triangular: Red). T.cf.panhai sp. I (Square: Yellow). T.cf.panhai sp. II (Circle: Green). *T.fujitanii* (Inverse triangular: Purple). *T.arduus*, *T.inornatus*, *T.pseudofrequentus* (Hexagon: Orange). *T.frequentus* (Star: Blue).

The results of this study provide a better understanding of the distribution of this genus, as Thailand is located in the middle of the distribution of other known Oriental species, but the genus was not reported from this area until this study (Fig. 6). We expect a broader distribution of the genus in Thailand, especially in the southern and eastern parts, as well as in rather poorly sampled areas, such as Myanmar, continental Malaysia, Laos, Cambodia, and Vietnam.

### ﻿Key to Oriental species of the genus *Tenuibaetis*

**Table:** 

1	Hindwing pads present (Müller-Liebenau and Hubbard 1985, fig. 1, p. 540)	**2**
–	Hindwing pads absent (Fig. 3A). Denticles on both mandibles pointed, with deep groove between denticle; Length of maxillary palp vs. galea-lacinia about 1.4–1.5×; about 8 setae along tibia-patella suture on tibia (Figs 2B, 2D, 2G, 3B)	***T.panhai* sp. nov.**
2	Labrum without U-shaped dark brown pattern, Gills without or with poorly developed tracheation (Kang et al. 1994, fig. 11A, L, p. 27; Kubendran et al. 2015, figs 4 (p. 190), 13–14 (p. 191))	**3**
–	Labrum with U-shaped dark brown pattern; Gills with developed tracheation (Kang et al. 1994, fig. 13A, K, p. 30)	***T.inornatus***
3	Abdominal tergites with distinct pattern coloration (Kang et al. 1994, figs 25, 27, p. 42; Kubendran et al. 2015, figs 1–2, p. 190); spines at posterior margin of terga mostly triangular (Kang et al. 1994, figs 11K, 14L, p. 27; Kubendran et al. 2015, fig. 16, p. 191)	**4**
–	Abdominal tergites rather uniform brown; spines at posterior margin terga mostly rounded (Kaltenbach and Gattolliat 2019, figs 1 (p. 16), 3c (p. 19))	***T.fujitanii***
4	Dorsal margin of tibiae and tarsi with short spine-like setae (Müller-Liebenau and Hubbard 1985, fig. 1g, 540). Spines at posterior margin of terga mostly triangular pointed, longer than wide (Kang et al. 1994, fig. 11K, p. 27; Kubendran et al. 2015, fig. 16, p. 191); Length of terminal filament vs. cerci about 0.6× (Müller-Liebenau and Hubbard 1985, fig. 1i, p. 540; Kubendran et al. 2015, figs 1–2, p. 190)	**5**
–	Dorsal margin of tibiae and tarsi with only thin setae. Spines at posterior margin of terga mostly triangular blunt, wider than long (Kang et al. 1994, fig. 14L, p. 31); Length of terminal filament vs. cerci 0.75×	***T.arduus***
5	Length of gill IV 2.0× of gill I; posterior margin of paraproct with about 20 spines (Müller-Liebenau and Hubbard 1985, fig. 1h, j, p. 540; Kubendran et al. 2015, fig. 15, p. 191)	***T.frequentus***
–	Length of gill IV 2.7–3.0× of gill I; posterior margin of paraproct with about 10 spines (Kang et al. 1994, fig. 11I, L, p. 27)	***T.pseudofrequentus***

## Supplementary Material

XML Treatment for Tenuibaetis panhai
